# The application of ultrasound-guided cannulated screw removal after proximal tibial fracture surgery: a retrospective study

**DOI:** 10.3389/fped.2025.1535675

**Published:** 2025-04-15

**Authors:** Kai Gu, Jiaqiang Qin, Meizhen Guo, Ruiliang Chu, Jiaxu Chen, Fusheng Qian, Yi Zhong

**Affiliations:** ^1^ Department of Orthopedics, Chongqing Key Laboratory of Pediatrics, Ministry of Education Key Laboratory of Child Development and Disorders, National Clinical Research Center for Child Health and Disorders, China International Science and Technology Cooperation Base of Child Development and Critical Disorders, Children’s Hospital of Chongqing Medical University, Chongqing, China; ^2^ Big Data Engineering Center (Medical Records Management Center), Chongqing Key Laboratory of Pediatrics, Ministry of Education Key Laboratory of Child Development and Disorders, National Clinical Research Center for Child Health and Disorders, China International Science and Technology Cooperation Base of Child Development and Critical Disorders, Children’s Hospital of Chongqing Medical University, Chongqing, China; ^3^ Department of Ultrasonography, Chongqing Key Laboratory of Pediatrics, Ministry of Education Key Laboratory of Child Development and Disorders, National Clinical Research Center for Child Health and Disorders, China International Science and Technology Cooperation Base of Child Development and Critical Disorders, Children’s Hospital of Chongqing Medical University, Chongqing, China

**Keywords:** bone screws, ultrasound, child, minimally invasive surgery, cannulated bone screw

## Abstract

**Introduction:**

Cannulated screws are widely used in pediatric orthopedic fixation. However, traditional screw removal requires preoperative x-ray positioning, leading to exposure to ionizing radiation. Sometimes, a larger incision is required, and trauma is a significant cause of x-ray positioning, which cannot be accurately marked on the skin. In this study, we aimed to evaluate the practicability of rapid removal of cannulated screws through a guide needle and small incision using ultrasound (US) guidance to locate the position of the screws accurately.

**Methods:**

A retrospective analysis was performed on patients who underwent removal of internal fixation after proximal tibial fracture surgery at our hospital between January 2019 and March 2024. Patients were divided into Group A and Group B based on the different cannulated screw removal techniques. In Group A, the cannulated screws were removed under US guidance, while in Group B, they were removed under direct visualization using a traditional incision. The operative time, blood loss, success rate of removal, radiation frequency, and incision length were statistically analyzed. Knee function was evaluated using the Knee Society Score.

**Results:**

53 patients aged 15.3 ± 0.1 years were included in this study. Group A showed 50% shorter incision length (1.5 vs. 3.0 cm, *p* = 0.005) and average screw incisions (0.5 vs. 1.0 cm, *p* = 0.007), along with complete elimination of preoperative radiation exposure (0 vs. 2 times, *p* < 0.001) and dose (0 vs. 0.102 mGy, *p* < 0.001) compared to Group B. Participants in Group A had no postoperative complications, whereas one patient in Group B had an incision infection; however, no significant difference was observed between the groups.

**Discussion:**

US-guided cannulated screw removal can be used in children with proximal tibial fractures, significantly reducing the preoperative radiation time and dose while minimizing the incisional length.

**Level of evidence:**

III

## Introduction

The cannulated screw can be easily placed percutaneously after closed reduction because it has a hollow structure that can be passed through the guide needle. It has been widely used in the fixation of pediatric fractures because it reduces patient trauma, such as femoral neck fracture (1, 2), tibial tuberosity fracture (3), epiphyseal fracture (4), and capitellum fracture (5). Metal implants can be selectively removed as they generally do not adversely affect the human body. However, experts recommend the removal of instrumentation because of concerns regarding the child's growth potential and life cycle (6). Additionally, internal fixation may cause pain, allergies, tissue irritation, and infection (7–10). The long-term presence of implants may result in stress concentration on the screw and stress shielding around the screw (11, 12). It may also lead to excessive trace metal content in the human body owing to the electrochemical interaction of metals (13, 14). Therefore, most surgeons prefer to remove internal fixations in children postoperatively.

With the development of ultrasound (US), its applications in orthopedics have increased (15), including screening for developmental dysplasia of the hip (DDH) (16, 17), tendon injury evaluation (18–20), fracture diagnosis (21), and intraoperative localization of foreign bodies in the body (22). A strong echo is observed when the ultrasonic waves hit the metal. Theoretically, the projection of the screw tail on the cortical bone surface onto the skin can be located using US. Chen et al. (22) successfully removed foreign bodies using US guidance combined with methylene blue labeling, thus reducing the radiation time, wound length, and operative time. Su et al. (23) performed a US-guided method for the closed reduction of radial neck fractures, reducing x-ray exposure and operative time. Chen et al. (24) diagnosed pain from excessive screw length in adults following total hip replacement using US. According to the literature reviewed, no studies have investigated US-assisted removal of cannulated screws in children.

In this study, we aimed to evaluate the advantages of US guidance in the postoperative removal of cannulated screws through a small incision compared to traditional surgery in children with tibial fractures.

## Material and methods

### Patients

A retrospective analysis was performed on children who underwent the removal of proximal tibial cannulated screw internal fixation at our hospital between January 2019 and March 2023. The cohort was stratified into group A and B according to the surgical approach for screw removal. Group A was treated with a small US-guided incision, whereas Group B was treated with a traditional incision.

The inclusion criteria were: (1) proximal tibial fractures fixed using cannulated screws, (2) age <18 years, (3) fresh fractures, and (4) complete fracture union. The exclusion criteria were: (1) simultaneous use of other types of internal fixation materials, (2) incomplete follow-up data, (3) pathological fractures, and (4) poor fracture healing. Written informed consent was obtained from all participants or their legal guardians for the publication of any potentially identifiable images or data included in this article. This study was approved by the our hospital's Ethics Committee (approval number: 2024−152) and was conducted in accordance with relevant guidelines and regulations.

### Surgical procedures

All operations were performed by four attending doctors and one US localization doctor in our hospital. The US equipment used was produced by Mindray (TE9S; Shenzhen, China), with a probe model of L14-6 s and a frequency of 10 MHz.

**Figure 1:**
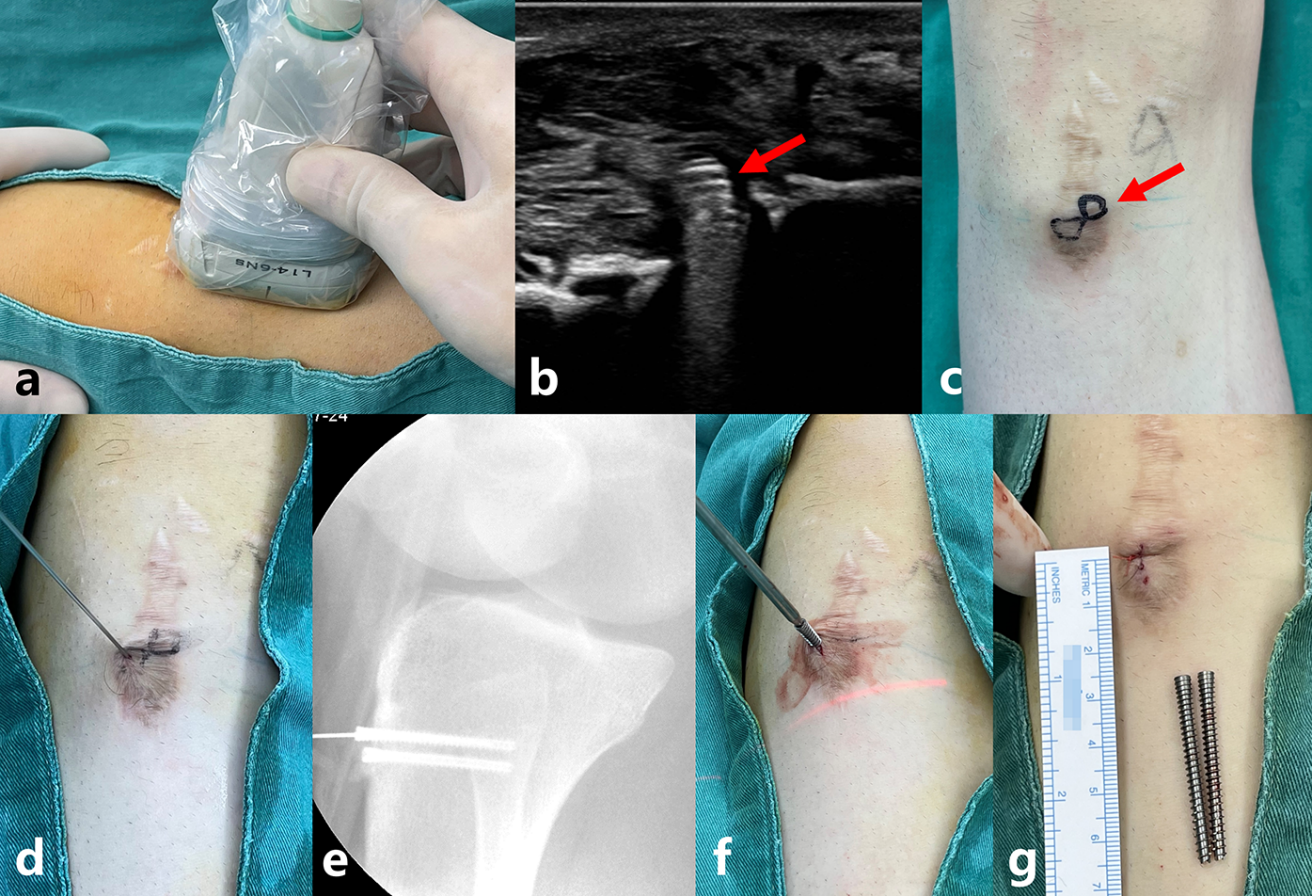
Shows a typical patient. A 13-year-old boy who came to remove the cannulated screws 14 months after right tibial tuberosity fracture surgery. He was injured because he tripped while running. **(a)** shows the position of the probe on the body surface. **(b)** shows the ultrasound image of the screw. The red arrow shows the screw tail. **(c)** shows the surface position of the screws. The red arrow shows the mark of the screw tail on the skin. **(d)** shows the penetration of the guide needle into the cannulated screw. **(e)** shows a radiograph confirming that the guide needle is inside the cannulated screw. Radiographs are not required after becoming proficient in surgical operations. **(f)** shows the placement of the screwdriver along the guide needle and the removal of the screw. **(g)** shows the incision being only 0.5 cm, and the screws were successfully removed.

Patients in Group A were scanned using a US probe near the incision at screw penetration before disinfection. The position of the screw tail was determined using a strong echo (Figure 1b), and its projected position on the skin surface was marked with a marker pen. After disinfection, a screw-guide needle with an appropriate diameter was inserted into the skin at the surface positioning center of the screw tail. The tail of the screw (metal friction) was detected. The tunnel of the cannulated screw was fully penetrated by shaking the needle from side to side and slightly adjusting the direction. Third, a small incision was made at the skin entry needle. The skin and subcutaneous tissues were separated using hemostatic forceps up to the tail of the screw. Finally, a cannulated screwdriver was inserted along the guide needle, the screw was removed by rotation, and the incision was stitched (Figure 1).

In Group B, the protrusion of the screw tail was marked by palpation before disinfection. If the position of the screw tail could not be determined by palpation, posterior marking was confirmed using x-ray fluoroscopy. After disinfection and coverage with a sterile towel, the skin and subcutaneous tissue were cut along the original incision, screws were removed, and the incision was sutured. In patients in whom the screw deviated far from the incision, it was removed after a skin incision at the surface.

Postoperative evaluation: The operative time, average screw removal time, incision length, average screw incision length, number of internal fixations, blood loss, radiation time and dose before screw removal, hospital stay, and cost were recorded using the medical data of each group. The operative time encompassed both the US imaging and screw removal procedures. The mean screw removal time was calculated by dividing the operative time by the number of screws. The average screw incision length was calculated by dividing the incision length by the number of screws used. The wound condition was monitored within 5 days after surgery. The Knee Society Score was used to evaluate knee function 1 month after surgery. According to the score, they were divided into two groups: excellent (≥85 points) and poor (<85 points) (25).

### Statistical analysis

All data were analyzed using SPSS 26.0 (IBM Corp., Armonk, NY, USA). Non-normally distributed data were expressed as median and quartile. Continuous variables were tested using the non-parametric Mann–Whitney *U*-test, while categorical variables were tested using the chi-square test. *P* < 0.05 was considered statistically significant.

## Results

### Patient characteristics

The study included 53 patients with a mean age of 15.3 ± 0.1 years, 10 in Group A, aged 15.1 (14.6–16.2) years, and 43 in Group B, aged 15.3 (14.5–15.9) years. Age did not significantly differ between the two groups.

### Radiation exposure analysis

Group A had a preoperative radiation time of 0 min, which was significantly lower than that of Group B (*P* < 0.001). The preoperative radiation dose in group A was 0 mGy, which was significantly lower than that in group B (*P* < 0.001).

### Surgical outcomes

The average incision length of Group A was 1.5 (1–2.25) cm, which was shorter than that in Group B (*P* = 0.005). The average screw incision length in Group A was 0.5 (0.46–1) cm, which was shorter than that in Group B (*P* = 0.007). All incisions in Group A healed well, and one patient in Group B showed redness and swelling. No statistically significant differences were observed between the two groups in terms of operative time, mean screw removal time, blood loss, hospitalization days, and hospitalization cost (Table 1). Despite the higher localization costs in Group A compared with Group B, the total hospitalization costs for patients showed no significant difference between the two groups.

**Table 1:** Patients’ demographic characteristics, surgery evaluation, hospitalization costs, complications and KSS.

Demographic and clinical characteristics	Group A	Group B	*P*-value
Age（yrs）	15.1 (14.6–16.2）	15.3 (14.5–15.9）	0.785^a^
Surgical site			
Left	2	25	
Right	7	17	
Bilateral	1	1	0.061^b^
Number of screws			
Left	5	75	
Right	20	48	
Bilateral	5	6	<0.001^b^
Total	30	129	
Average number of screws	3 (2.75–3）	3 (3–3）	0.886^a^
Number of washers			
Left	3	21	
Right	9	4	
Bilateral	0	0	
Total	12	25	0.002^b^
Surgical outcomes			
Operation time（min）	35（25–60）	35.5（20.25–51）	0.459^a^
Average screw removal time（min）	10.87 (7.46–17.25）	13.75 (10–18.33）	0.295^a^
Preoperative radiation times	0	2 (1–1）	<0.001^a^
Preoperative radiation dose (mGy)	0	0.102（0.095–0.135）	<0.001^a^
Incision length（cm）	1.5 (1–2.25）	3 (2–4）	0.005^a^
Average screw incision length（cm）	0.5 (0.46–1）	1 (0.75–1.33）	0.007^a^
Blood loss during surgery（ml）	2.5 (2–5）	3 (1–5）	0.808^a^
Cost			
costs of localization (￥)	80.0	30.0	<0.001^a^
hospitalization days (d)	6.5 (5.75–7.25）	6 (5–6）	0.116^a^
hospitalization cost (￥)	6,848.1 (6,323.6–7,585.0）	7,013.3 (6,267.1–7,536.8）	0.838^a^
Complications	0	1^c^	1^b^
KSS			
Knee joint score	100	100	0.946^b^
Activity function rating	100	100	1^b^

KSS, Knee Society Score.

Descriptive statistics are presented as medians and interquartile ranges for continuous variables and as counts for categorical variables.

^a^ Indicates the chi-square test for categorical variables.

^b^ Indicates the Mann–Whitney *U*-test for continuous variables. Statistical significant was set at *P* < 0.05.

^c^ The incision appeared incision redness and swelling. It finally healed after continuous dressing changes.

## Discussion

This study proved that US-guided removal of cannulated screws in the proximal tibia effectively reduces radiation time, radiation dose, and surgical incision compared to traditional surgery.

**Figure 2:**
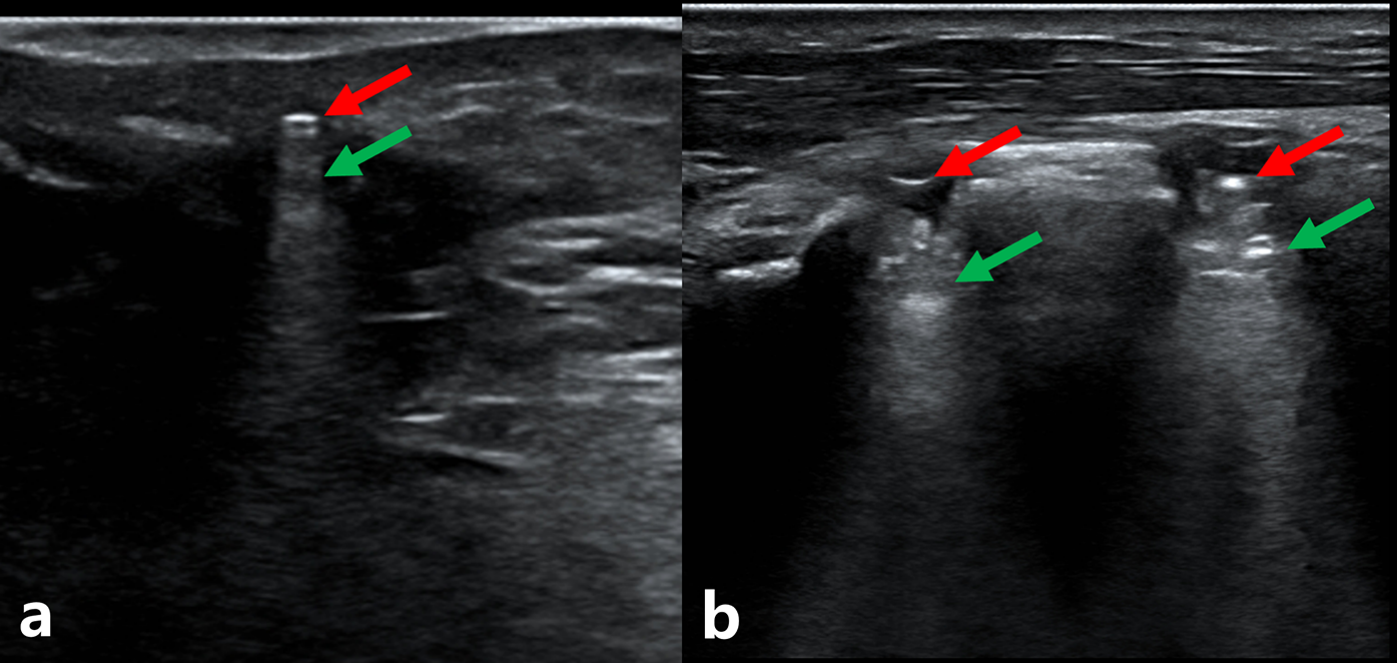
Displays the ultrasound images of a 15-year-old boy with three cannulated screws in his right tibia. **(a)** shows one of the screws. **(b)** shows the other two screws in one scan. The red arrow shows that the strong echo produced by the ultrasonic hits the screw tail. The green arrow refers to the acoustic shadow below the screw tail.

US, as a non-invasive and radiation-free imaging modality with rapid scanning capability, is unlikely to induce thermal tissue damage (26, 27). To the best of our knowledge, no previous studies have investigated the adverse effects of US. US can dynamically observe the morphology of bone and muscle tissues. Numerous studies have reported its wide application in orthopedics. For example, Dong et al. (16) and Xiao et al. (17) identified the risk factors for DDH while using US in DDH diagnosis. They believed that US played an important role in the early diagnosis, treatment, and follow-up of DDH. Snelling et al. (21) confirmed that US had high accuracy in diagnosing fractures in 135 children with distal forearm injuries. A previous study (28) reported that US is a highly sensitive and accurate tool for detecting radiolucent foreign bodies. Jia et al. (29) reported using US-guided closed reduction and internal fixation for ulnar and radial fractures in children, which could reduce radiation dose and operative time. Su et al. (23) reported US-guided treatment of pediatric radial neck fractures. US can help avoid injury to the posterior interosseous nerve of the radius by providing a precise location. When ultrasonic waves hit metal, they produce a strong echo with accompanying acoustic shadows (Figure 2). Chen et al. (24) reported two patients experiencing pain after total hip replacement. They found an acetabular screw protruding from the ischium on US examination, and the pain significantly improved after screw removal. Therefore, US localization of screws *in vivo* is feasible. To date, we have not found any studies related to the minimally invasive removal of cannulated screws after US positioning.

Radiation exposure and damage in children cannot be ignored (30–34). We concur with the principle that radiation exposure should be minimized during pediatric surgery (35), even though the dose of radiation that causes cancer is currently controversial. According to Su et al. (23), the fracture site of the radial neck and Kirschner wire can be dynamically observed using US guidance. Using US guidance for the closed reduction of radial neck fractures in children can significantly reduce the time and dose of radiation compared to reduction under x-ray fluoroscopy. Shen et al. (36) reported a method for closed reduction of lateral humeral condyle fractures in children assisted by US without intraoperative x-ray fluoroscopy and achieved satisfactory results in fracture reduction and elbow joint function. Various methods exist to locate the screw tail during cannulated screw removal; for example, the screw tail can be palpated on the body surface. However, most tibial tubercles are in athletic adolescents with thick subcutaneous fat, and it is difficult to touch the screw tail accurately by palpation. Sometimes, misjudgment may occur because of blocking of the periosteum or scarring, rendering the method inaccurate. Multi-angle fluoroscopy, often required 1–3 times, facilitates accurate positioning because fluoroscopy produces a planar image. In this study, the US positioning method was used to dynamically scan the cortical bone of the surgical area, and the screw tail could be accurately located according to the “strong echo” and reduced radiation exposure.

Accurate localization of the surgical site can shorten the surgical incision. Chen et al. (22) documented US-guided removal of foreign bodies on the body surface. Using US to locate the foreign body, methylene blue was injected around the foreign body for labeling under dynamic US monitoring. The incision length in 11 patients was 1.21 ± 0.13 cm, significantly shorter than that of the control group. Li et al. (37) reported on 85 patients who underwent long tubular bone internal fixation removal, with 40 patients undergoing minimally invasive methods resulting in an average incision length of 5.07 ± 1.18 cm, significantly shorter than that of the traditional group. However, x-ray fluoroscopy is required during surgery, and metal plates are used for internal fixation material. As mentioned previously, locating screws at superficial sites during cannulated screw removal can be challenging. Traditional screw removal methods involve cutting the original scar and removing the screw under direct visualization. The surgery is simple; however, the incision is long. Sometimes, repeated exploration is necessary to locate the screw, or the incision may need to be extended if the screw placement point is far from the incision. Traditional surgery may require a larger incision than previous surgery, which undoubtedly increases surgical trauma. The method used in this study reduced surgical trauma. The screw was removed under US guidance, and the average screw incision length was only 0.5 cm, equivalent to the diameter of the cannulated screw tail and significantly shorter than that of the control group. This reduction is attributed to precise incision placement after determining the position of the screw tail using US, thus avoiding prolongation of the incision owing to inaccurate positioning to reduce trauma.

A guide needle was used to probe the screw to determine its position. The specific method is to use a guide needle to “scan” the puncture near the screw. When metal friction was felt, the direction was adjusted by slight shaking, and the central tunnel of the screw was inserted. A small incision was made at the skin puncture site, and the screw was removed using a matching cannulated screwdriver under the guidance of a guide needle. This method involves a small incision and can accurately locate the position of the screw; multiple punctures are sometimes required to locate the tail of the screw, which requires more time and increases acupuncture trauma.

Less tissue dissection results in less bleeding. No significant difference was observed in the amount of bleeding between the two groups in this study. Since the study population included patients with proximal tibial fractures, we used a tourniquet during surgery. The only bleeding event that occurred during the operation involved oozing blood from the nail tract, and the amount of bleeding was low. According to Li et al. (37), the internal fixation of the metal plate was removed through a minimally invasive incision. The intraoperative and postoperative blood loss were 103.10 ± 24.44 ml and 44.30 ± 10.93 ml, respectively, significantly less than that of traditional incision removal of internal fixation. This reduction is because a minimally invasive approach reduces the incision length and exposes less tissue. We believe that in patients in whom a tourniquet is missing or cannot be used, such as those with cannulated screws at the femoral neck, bleeding will be reduced owing to smaller wounds relative to traditional open removal.

This method is considered feasible for the removal of deep cannulated screws, including those after femoral neck fracture. US techniques can guide the insertion of a guide needle into the interior of the cannulated screw and remove the internal fixation through a small incision. This approach avoids excessive exposure of the fascia lata and deep tissues. Theoretically, it can reduce the incision, amount of bleeding, and suture time.

Certain difficulties may be encountered during the operation. First, the screw tail is covered by soft tissue, which causes the driver to fail to engage smoothly. However, under the guidance of a guide needle, the screwdriver was gently rotated to the left and right when it reached the screw. Simultaneously, pressure was applied to extrude the soft tissue and engage the screwdriver with the nut. Second, removing screws using washers is more difficult; it requires a detailed preoperative review of the medical records of the previous operation and a careful examination of the radiographs to determine the presence of the washer. In patients with washers, we probed the washer deep along the screw using a vascular clamp or stripper before completely removing the screw. Subsequently, the washer and the screw was removed.

Our study had some limitations. First, the learning curve is long, requiring surgeons to have imaging knowledge and the ability to recognize US images. Young residents may not be able to fully grasp the direction of the guide needle, resulting in a situation in which the guide needle cannot locate the screw tail or becomes bent. Based on our experience, it took approximately 5–10 cases for the surgical team to achieve proficiency, making the screw removal time using US equivalent to that using fluoroscopy. Second, the sample size was small; hence, more patients from multiple centers are required for verification. Third, this study was retrospective; thus, further prospective double-blind controlled studies are required. Lastly, the participants were inconsistent, resulting in differences in the timing of the surgical procedure.

## Conclusion

US-guided cannulated screw removal can significantly reduce the amount of preoperative radiation, radiation dose, and length of incision. It is an optional surgical method.

## Data Availability

The raw data supporting the conclusions of this article will be made available by the authors, without undue reservation.
